# Metabolic Biomarkers for the Early Detection of Cancer Cachexia

**DOI:** 10.3389/fcell.2021.720096

**Published:** 2021-09-21

**Authors:** Thomas M. O’Connell, Lilian Golzarri-Arroyo, Fabrizio Pin, Rafael Barreto, Stephanie L. Dickinson, Marion E. Couch, Andrea Bonetto

**Affiliations:** ^1^Department of Otolaryngology – Head and Neck Surgery, Indiana University School of Medicine, Indianapolis, IN, United States; ^2^Indiana Center for Musculoskeletal Health, Indiana University School of Medicine, Indianapolis, IN, United States; ^3^Simon Comprehensive Cancer Center, Indiana University School of Medicine, Indianapolis, IN, United States; ^4^IUPUI Center for Cachexia Research Innovation and Therapy, Indiana University School of Medicine, Indianapolis, IN, United States; ^5^Department of Epidemiology and Biostatistics, School of Public Health, Indiana University, Indianapolis, IN, United States; ^6^Department of Anatomy, Cell Biology and Physiology, Indiana University School of Medicine, Indianapolis, IN, United States; ^7^Department of Surgery, Indiana University School of Medicine, Indianapolis, IN, United States

**Keywords:** metabolomics, cancer cachexia, pre-cachexia, early detection, biomarkers, muscle wasting

## Abstract

**Background:** Cancer cachexia is a severe metabolic disorder characterized by progressive weight loss along with a dramatic loss in skeletal muscle and adipose tissue. Like cancer, cachexia progresses in stages starting with pre-cachexia to cachexia and finally to refractory cachexia. In the refractory stage, patients are no longer responsive to therapy and management of weight loss is no longer possible. It is therefore critical to detect cachexia as early as possible. In this study we applied a metabolomics approach to search for early biomarkers of cachexia.

**Methods:** Multi-platform metabolomics analyses were applied to the murine Colon-26 (C26) model of cachexia. Tumor bearing mice (*n* = 5) were sacrificed every other day over the 14-day time course and control mice (*n* = 5) were sacrificed every fourth day starting at day 2. Linear regression modeling of the data yielded metabolic trajectories that were compared with the trajectories of body weight and skeletal muscle loss to look for early biomarkers of cachexia.

**Results:** Weight loss in the tumor-bearing mice became significant at day 9 as did the loss of tibialis muscle. The loss of muscle in the gastrocnemius and quadriceps was significant at day 7. Reductions in amino acids were among the earliest metabolic biomarkers of cachexia. The earliest change was in methionine at day 4. Significant alterations in acylcarnitines and lipoproteins were also detected several days prior to weight loss.

**Conclusion:** The results of this study demonstrate that metabolic alterations appear well in advance of observable weight loss. The earliest and most significant alterations were found in amino acids and lipoproteins. Validation of these results in other models of cachexia and in clinical studies will pave the way for a clinical diagnostic panel for the early detection of cachexia. Such a panel would provide a tremendous advance in cachectic patient management and in the design of clinical trials for new therapeutic interventions.

## Introduction

It has been estimated that up to 80% of all patients with advanced cancer will be afflicted with the severe wasting syndrome known as cancer cachexia (Tisdale, 2002; Fearon et al., 2011). This multifactorial syndrome is characterized by progressive body weight loss accompanied by a pernicious depletion of skeletal muscle mass, with or without the loss of adipose tissue (Evans et al., 2008). The pathophysiology involves a negative protein and energy balance driven by inflammation and aberrant metabolism. Although cachexia is often accompanied by anorexia, it is distinct from starvation and cannot be reversed by nutritional interventions (Fearon et al., 2011). This progressively worsening condition leads to functional impairment and a reduced quality of life. In addition, cachexia can reduce patient’s ability to tolerate and respond to chemotherapy, sometimes leading to the inability to complete their therapeutic regimen (Wheelwright et al., 2013).

The lack of a set of clear and quantitative parameters to diagnose and classify cachexia has been a significant impediment in the clinical management of cachexia as well as in the development of clinical trials for new therapeutic approaches. In 2011, an international consensus defined the diagnostic criterion for cachexia as “weight loss greater than 5% or weight loss greater than 2% in individuals already showing depletion according to current body weight and height [body-mass index (BMI) < 20 kg/m^2^] or skeletal muscle mass (sarcopenia)” (Fearon et al., 2011). Furthermore, an agreement was made that cachexia, like cancer, can progress through stages starting with pre-cachexia, moving to cachexia and finally refractory cachexia. According to this progression, in the pre-cachexia stage patients have not lost more than 5% of their body weight, although they may show clinical and metabolic signs such as anorexia and impaired glucose tolerance. Once the weight loss has met the diagnostic criteria above, the patients have entered the cachectic stage. In the final, refractory cachexia stage, patients are in the advanced stages of cancer where they are no longer responsive to therapy and are experiencing aggressive hyper-catabolism such that any weight-loss management is no longer possible.

Given the critical role of metabolic dysregulation in the development and progression of cachexia, there has been an increasing application of metabolomics in the search for new biomarkers. Early metabolomics studies using the murine Colon-26 (C26) colorectal cancer model of cachexia found that the serum metabolome was characterized by alterations in glucose and lipid metabolism (O’Connell et al., 2008). In a subsequent study from our group using the same model, metabolomics revealed that the metabolic alterations in cachexia are distinct from those that accompany starvation or tumor burden (Der-Torossian et al., 2013). To further define mechanisms, several studies have examined tissue samples in addition to blood. Using nuclear magnetic resonance (NMR)-based metabolomics on serum along with solid state, magic angle spinning NMR on muscle tissue, Yang et al. (2018), found that cachexia could be characterized by five metabolic “hubs” including low blood glucose, elevated ketone bodies, decreased branched chain amino acids (BCAAs), increased neutral amino acids and high 3-methylhistidine and creatine. This study included longitudinal sampling to look for early biomarkers of cachexia, i.e., at the pre-cachexia stage, but supervised partial least squares (PLS) analysis of the data showed that the profiles from the early time points were clustered closely with the control group. In an analysis of serum and muscle tissue using GC/mass spectrometry (MS), Lautaoja et al. (2019), reported alterations in amino acids, energy metabolites, and nucleotides in serum along with altered amino acids in muscle. In particular they found that free phenylalanine strongly correlated with the loss of body mass.

We conducted a metabolomics investigation of cachexia induced by cancer and chemotherapy which included serum, muscle, and liver tissue (Pin et al., 2019). These studies found some common and some distinct metabolic differences in cachexia induced by cancer versus chemotherapy, in line with previous molecular observations (Barreto et al., 2016). The discriminatory metabolites included those involved in glycolysis, lipid, and fatty acid metabolism.

In human studies, most metabolomics investigations have focused on biomarkers in easily obtainable biofluids. In a study of 93 cancer patients, Eisner et al. (2011), evaluated the urinary metabolome to detect cancer associated muscle wasting. Metabolite profiles were correlated with muscle wasting determined by changes in computed tomography (CT) of the lumbar skeletal muscle area. Machine learning techniques yielded a predictive accuracy of 82.2%.

In a study of serum metabolite profiles from cachectic patients Cala et al. (2018), utilized three MS-based analytical platforms and found that decreases in amino acids, glycerophospholipids, and sphingolipids were associated with cachexia. Consistent with this study, Miller et al. (2019), also applied a MS-based analysis of serum and found that alterations in amino acid and lipid metabolites were highly discriminative of weight loss.

Using NMR-based metabolomics on both serum and urine from patients with a range of cancers, Yang et al. (2018), used a logistic regression to develop a diagnostic model based on three metabolites, carnosine, leucine, and phenylacetate. The corresponding ROC curve had an AUC of 0.991 and the model had a predictive accuracy of 94.6%. Interestingly, this cohort included patients designated as weight stable, pre-cachectic, and cachectic, but no predictive models were generated to specifically identify patients in the pre-cachectic phase. Ose et al. (2019), also looked for biomarkers of pre-cachexia in a cohort of colorectal cancer patients classified as cachectic (*n* = 16), pre-cachectic (*n* = 13), and non-cachectic (*n* = 23). Using both untargeted GC/MS and NMR they found that cachectic patients were characterized by higher levels of acetone and arginine. No distinction between pre-cachectic and cachectic was reported.

In order to develop a metabolic biomarker panel for the detection of cachexia in the early stages, we have applied a multi-platform metabolomics strategy to profile the progressive changes in the metabolome in the murine C26 colorectal tumor model of cachexia. In this study, serum was analyzed using three platforms including untargeted NMR, targeted MS, and NMR-based lipoprotein profiling. This set of platforms was selected to provide a uniquely broad coverage of the metabolome. In contrast to many untargeted metabolomics approaches, the three platforms that we applied all yield quantitative data. The NMR platforms are inherently quantitative, and the MS platform utilized a set of calibration standards to insure quantitative results. Another unique feature of this study is the high-resolution, longitudinal study design to examine the detailed trajectory of cachectic progression. This enabled a more detailed analysis of the metabolic perturbations beyond what would be detected by simply examining samples defined as pre-cachectic and cachectic. The data show that some metabolic perturbations are detectable well in advance of the observation of weight loss pointing to the possibility that metabolic biomarkers may be useful to detect cachexia early, potentially in the pre-cachexia stage.

## Materials and Methods

### Animals

All animal experiments were conducted with the approval of the Institutional Animal Care and Use Committee at Indiana University School of Medicine and were in compliance with the National Institutes of Health Guidelines for Use and Care of Laboratory Animals and with the ethical standards laid down in the 1964 Declaration of Helsinki and its later amendments. For this study we used 8-week old CD2F1 male mice (Harlan, Indianapolis, IN, United States). The animals were maintained on a regular dark-light cycle (light from 8 AM to 8 PM), with free access to food and water during the whole experimental period. All mice were fed Teklad 2018X grain-based chow. The temperature in the room was set to 22 ± 2°C and the relative humidity was maintained between 30 and 70%. Mice were divided randomly into two groups: mice inoculated with vehicle served as controls (*n* = 20) and tumor-bearing animals injected intrascapularly (s.c.) with 1 × 10^6^ C26 adenocarcinoma cells in sterile saline (*n* = 35). Animals were monitored and weighed daily until the day of sacrifice. A set of three animals in the control group were sacrificed on days 2, 6, 10, and 14. A set of three tumor-bearing animals were sacrificed on days, 2, 4, 6, 8, 10, 12, and 14. Skeletal muscles (tibialis anterior, gastrocnemius, and quadriceps) were collected, weighed, snap frozen in liquid nitrogen, and stored at –80°C for further studies. Total blood was withdrawn from anesthetized mice by cardiac puncture and collected in EDTA-containing tubes. To separate plasma from the hematocrit, all the blood was centrifuged for 15 min at 3500 rpm.

### Cell Lines

Murine C26 cells were provided by Donna McCarthy (Ohio State University) and cultured in high glucose (4.5 g/L) Dulbecco’s Modified Eagle’s Medium (DMEM) supplied with 10% fetal bovine serum, 1% glutamine, 1% sodium pyruvate, and 1% penicillin/streptomycin. Cell were maintained in a 5% CO_2_, 37°C humidified incubator.

### Sample Preparation for NMR

Plasma samples for NMR analysis were prepared diluting 100 μl of plasma with 500 μl of a deuterated phosphate buffer solution (pH = 7.4) containing 2,2-dimethyl-2-silapentane-5-sulfonate sodium salt (DSS) with a final concentration of 0.5 mM to be used as a chemical shift and quantitation reference. The solution was then filtered through a 10 KDa, molecular weight cutoff filter to remove the proteins. Samples were placed in 5 mm NMR tube for analysis.

### Sample Preparation for Mass Spectrometry

Samples for targeted MS analysis were conducted using the Biocrates Absolute IDQ kit (Biocrates, Innsbruck, Austria). Each plate contains 16 wells reserved for selected internal standards to optimize the metabolite quantification. For serum analysis, 10 μl aliquots were loaded directly into the 96 well plate followed by derivatization and extraction per vendor protocols.

### NMR Data Collection

Nuclear magnetic resonance data were acquired on a Bruker Avance III 700 MHz NMR spectrometer with a TXI triple resonance probe operating at 25°C. Spectra were collected with a 1D NOESY pulse sequence covering 12 ppm. The spectra were digitized with 32,768 points during a 3.9 s acquisition time. The mixing time was set to 100 ms and the relaxation delay between scans was set to 2.0 s.

### NMR Lipoprotein Profiling

Determination of lipoprotein particle subfractions by NMR was assessed using the LipoScience Vantera platform using 125 μl of serum (LabCorp Inc., Burlington, NC, United States). Lipoprotein particle numbers and sizes were determined using a version of the LP2 algorithm (Jeyarajah et al., 2006) modified for murine plasma. GlycA levels were quantified as described by Otvos et al. (2015).

### NMR Data Processing

The data were processed using Advanced Chemistry Development Spectrus Processor (version 2016.1, Toronto, ON, Canada). The spectra were zero filled to 65,536 points, apodized using a 0.3 Hz decaying exponential function and fast Fourier transformed. Automated phase correction and third order polynomial baseline correction was applied to all samples. Metabolite concentrations were quantified using the Chenomx NMR Suite (version 8.2, Chenomx Inc., Edmonton, AB, Canada). The DSS-d_6_ was used as a chemical shift and quantification reference for all spectra and was set to a chemical shift of 0.00 and a concentration of 500 μM. Quantitative fitting of each spectrum was carried out in batch mode, followed by manual adjustment for some spectra to correct for errors arising from spectral overlap.

### Mass Spectrometry Data Collection

This Biocrates AbsoluteIDQ p180 assay quantifies 187 metabolites from five chemical classes: acylcarnitines, amino acids, biogenic amines, hexoses (sum of hexoses), phosphocholines (PCs), and sphingomyelins (SMs). Data were collected on an AB Sciex 4000 QTRAP coupled to an Acquity UPLC system with the selective mass-spectrometric detection using multiple reaction monitoring (MRM) pairs. The amino acids and biogenic amines were detected using and LC–MS/MS method and the lipid species were detected using a flow injection analysis (FIA) MS/MS method per vendor defined settings.

### Mass Spectrometry Data Analysis

Data analysis including normalization for quantification of metabolite concentrations and quality assessment were performed with the MetIDQ software package, which is an integral part of the AbsoluteIDQ kit. The metabolite concentration of each metabolite in in the series was compared with the measurement detection limit specifications as reported by the manufacturer of the AbsoluteIDQ p180 kit (Biocrates). A metabolite was excluded from further analyses if it was not present in more than 50% of the samples. Note that zero values for several of the lipoproteins were observed in the early timepoints, but these measurements were kept as the later day values showed significant differences.

### Statistical Analysis

For all data, an evaluation of the normality of the metabolite distribution was carried out to determine if a log transformation should be applied to the data. The skewness was calculated for the metabolites with and without log transformation and the distribution which had the skewness closest to zero was used in further analyses. The metabolites that were transformed have “_log” suffix. For each metabolite, a linear regression was modeled with a quadratic fit across time (days 2–14) including a factor for treatment group (C26, Control), and an interaction of group × time. Estimated means were produced from each model by treatment group, by day, using contrast statements, to see which metabolites showed differences between groups at specific days (6–14). Estimated means and standard errors were calculated for each metabolite on each day, *t*-tests and *p*-values comparing between groups were computed.

## Results

### Progressive Loss of Body Weight and Skeletal Muscle With Cachexia

**FIGURE 1:**
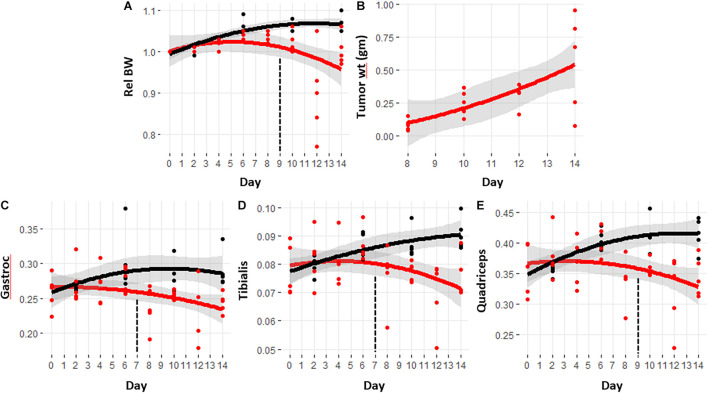
Trajectories of body weight, tumor size, and skeletal muscle masses with the progression of cancer-induced cachexia. Trajectories are calculated using a quadratic function with gray bands indicating the 95% confidence interval for the fit. Black lines are from control mice and red lines are from tumor hosts. Vertical dashed lines indicate the day in which the trajectories became significantly different. The *p*-values for this data are provided in Supplementary Table 1. **(A)** Relative body weight, **(B)** tumor size measured at sacrifice, **(C–E)** weights of gastrocnemius, quadriceps, and tibialis respectively, taken at sacrifice.

The well-established, murine C26 colorectal cancer model of cancer cachexia has a time course of approximately 13–14 days (Tanaka et al., 1990; Bonetto et al., 2016). The trajectory of relative body weight change for both the tumor hosts and controls is shown in Figure 1A. The control mice demonstrated an expected increase in body weight, whereas the weight trajectory of the tumor-bearing mice demonstrated significant reductions starting at day 9 (see Supplementary Table 1 for *p*-values). Figure 1B shows the increase in tumor size over the same time course. The tumor is usually palpable around day 6, but typically <5 mg in size, averaging about one tenth of the final tumor size. Figures 1C–E show the weight trajectories of the gastrocnemius, tibialis, and quadriceps. Significant differences between control and cachexia were observed at day 7 for the gastrocnemius and quadriceps and day 9 for the tibialis.

The relative body and muscle weight trajectories show significant variability in the later days, especially day 12. Several lower values in the body and muscle weights of the mice sacrificed at day 12 appear to suggest that those mice were more cachectic than those sacrificed at day 14. Supplementary Figure 1 shows the boxplots of the relative body weights along with the tumor and muscle weights for the cachectic mice at days 10, 12, and 14. ANOVA analysis with Tukey *post hoc* correction shows that the body weight for the mice sacrificed at day 12 are significantly lower than at day 10, but not significantly different than at day 14. Comparison of the muscle weights showed no significant differences in these final 3 days. The muscle weight data indicate that the mice at day 14 were experiencing a comparable state of cachexia to those sacrificed at the earlier timepoints. The reduced mean values of the muscle weights as observed for the tibialis and quadriceps may suggest increased wasting, but given the variability in this model, a greater number of mice would be required to confirm this.

#### Comparison of Metabolite Trajectories

**FIGURE 2:**
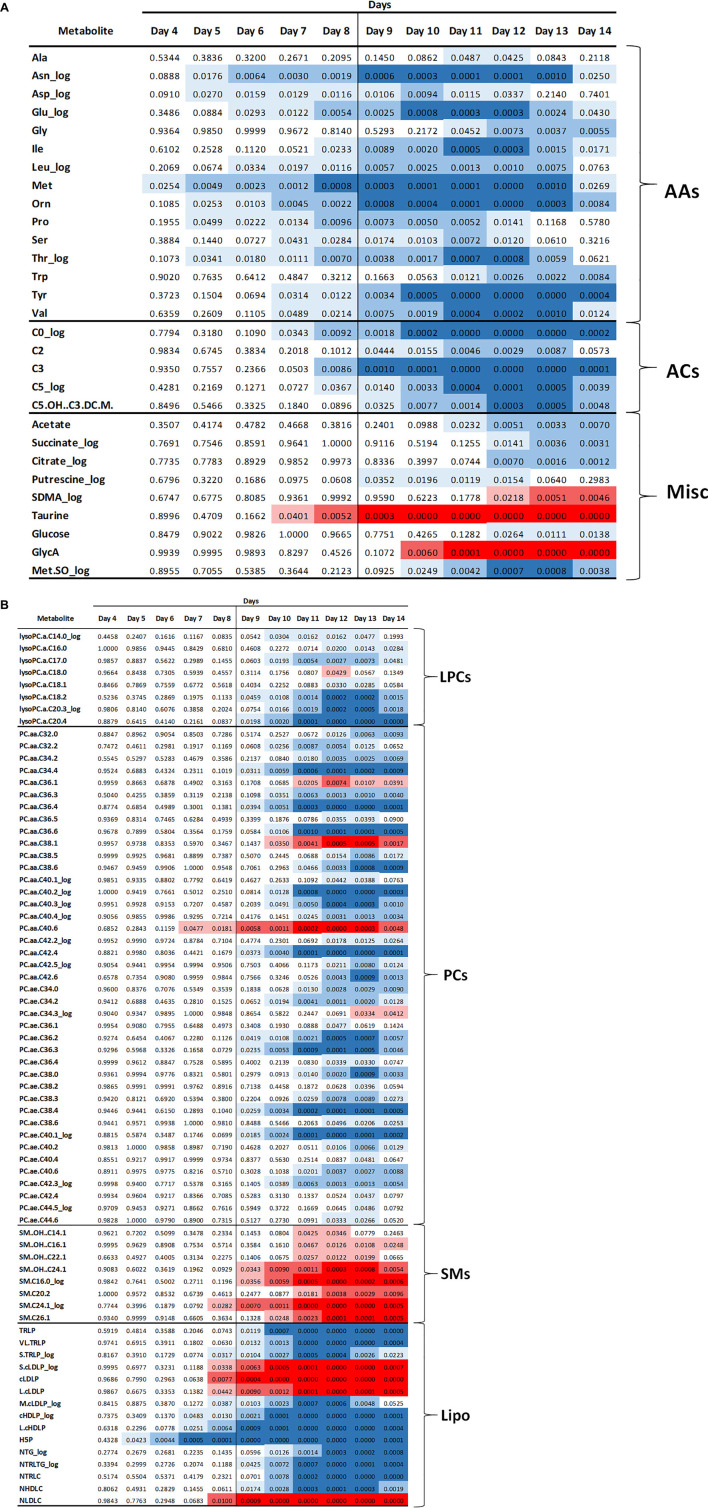
Heatmap of *p*-values for the significantly altered metabolites starting at day 4. Blue color indicates that the metabolite concentrations were reduced in the cachectic mice and red indicates that the concentrations were increased. Darker shading indicates more significant changes, i.e., lower *p*-values. **(A)** Data for amino acids (AA), acylcarnitines (AC), and miscellaneous metabolites (Misc). **(B)** Data for lipid related metabolites including lysophosphocholines (LPC), phosphocholines (PC), sphingomyelins (SM), and lipoproteins (Lipo).

Multi-platform metabolomics analyses were carried out on plasma samples from each of the mice over the time course of the experiment. The untargeted NMR analysis provided quantitative measurements of 18 metabolites, targeted MS platform measured 187 metabolites across five main chemical classes, including amino acids, organic acids, acylcarnitines, organic amines, and lipids. The lipoprotein profiling yielded particle concentrations for 21 species including triglyceride-rich particles, low-density particles, and high-density particles. Beyond lipoproteins, the LipoScience platform provided the inflammatory marker, GlycA which is an aggregate signal of acute phase proteins (Otvos et al., 2015). For each metabolite the skewness was calculated with and without log transformation and the dataset with a skewness closest to zero was used. As described in the methods, a linear regression model with a quadratic fit across days 2–14 including a factor for treatment group, and an interaction with time was computed. From this data the estimated means and standard errors of each metabolite at each day were computed; data shown in Supplementary Table 2. Each metabolite was analyzed for a statistically significant difference between the control and cachexia group at each day. Note that some metabolites were detected by multiple platforms. For consistency, if a metabolite was found to be significantly different by multiple platforms, the MS detected metabolites were used. The final analysis yielded 101 metabolites for which there was a significant difference between controls and cachexia in at least 1 day. Figure 2 shows heatmaps of the *p*-values for the significantly altered metabolites across days 4–14. These heatmaps illustrate that some metabolite changes manifest early while some manifest later and some increase through day 14 while others return to control levels in the later days.

### Amino Acids Are Early Biomarkers of Cachexia

**FIGURE 3:**
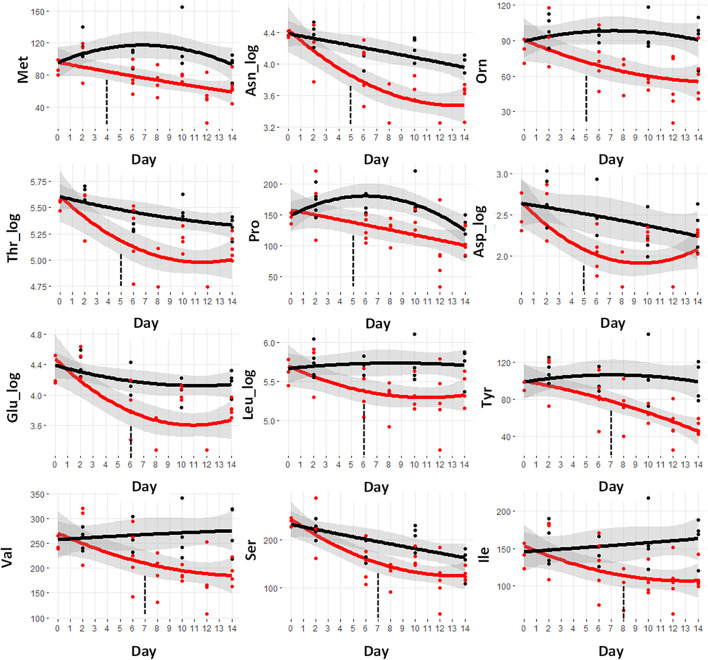
Trajectory of amino acid concentrations that were statistically significantly different prior to the observation of significant weight loss at day 9. Black indicates control mice, red indicates cachectic mice, and the vertical dashed lines indicate the day in which the data were first significantly different.

The trajectories of the amino acid concentrations were examined to look for those that demonstrated early differences. A set of 12 amino acids shows significant differences in the cachectic mice at or before day 8, prior to significant weight loss at day 9. Figure 3 shows the metabolite trajectories for each of these metabolites. The earliest metabolite to demonstrate a difference is methionine (Met) which is distinct in the cachectic mice starting at day 4 and going through day 14. A set of three amino acids shows differences starting at day 5 including asparagine, ornithine, threonine, proline, and aspartate. Only asparagine and ornithine maintain the differences through day 14 while threonine converges at day 13 and proline and aspartate converge at day 12. There are two amino acid measurements that are different at day 6, glutamate and leucine. The metabolites tyrosine, valine, and serine are different at day 7 and isoleucine is different at day 8. The pattern of convergence of some of these metabolites in the later days was unexpected but may suggest some competing mechanism between cachexia and the final stages of mortality. Overall, the amino acids represent the earliest detectable change in the serum metabolome.

### Progressive Reduction in Short Chain Acylcarnitines

Serum levels of acylcarnitines are a valuable metabolic surrogate for intermediates along the β-oxidation pathway. For fatty acid CoAs to enter the mitochondrion for oxidation, the CoA group is switched for a carnitine by the action of carnitine acyltransferases. The carnitine shuttle system transports the acylcarnitines into the mitochondria. The reverse reaction regenerates the acyl-CoA to enable the start of the β-oxidation process. The interconversion of acyl-CoA and acyl-carnitines continues and since the acylcarnitines can exit the cell, their serum concentrations reflect the progress of β-oxidation.

**FIGURE 4:**
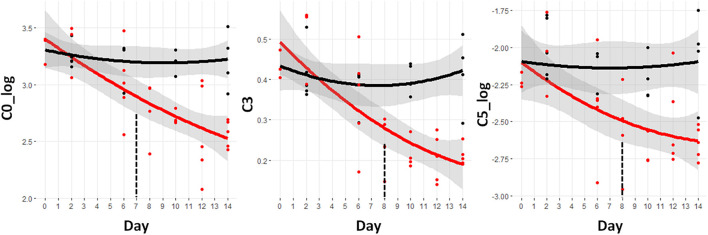
Trajectory of acylcarnitine concentrations that were statistically significantly different prior to the observation of significant weight loss at day 9. Black indicates control mice, red indicates cachectic mice, and the vertical dashed lines indicate the day in which the data were first significantly different. C0, free carnitine; C3, propionyl carnitine; C5, valerylcarnitine.

Figure 4 shows the trajectories of the three acylcarnitines that are significantly different prior to day 9. These are all short chain acylcarnitines. Free carnitine without an acyl group attached is designated C0. The log value of this metabolite demonstrates the earliest difference starting at day 7 and going through day 14. The C3 and C5_log species are reduced at day 8 and continue to drop through day 14. The C3 and C5 acylcarnitines can be derived from both fatty acids and BCAAs and so the drop in these species is consistent with the BCAA reductions shown in Figure 3.

#### Alterations in Lipoproteins

Lipoproteins are macromolecular aggregates in the blood that are used to shuttle hydrophobic lipids throughout the body. There are four major classes of lipoproteins; chylomicrons, very-low-density lipoproteins (VLDLs), low-density lipoproteins (LDLs), and high-density lipoproteins (HDLs). The chylomicrons are produced in the small intestines during fat absorption and are composed mainly of triglycerides. The VLDL particles are triglyceride-rich particles produced by the liver that are turned into LDL particles through the action of lipoprotein lipase (LPL). LDLs are the major cholesterol carriers in the blood and shuttle cholesterol to peripheral tissues for membrane synthesis or to the liver for cholesterol metabolism. HDL particles contain mainly phospholipids and a small amount of cholesterol that is obtained from peripheral tissue and transported back to the liver.

**FIGURE 5:**
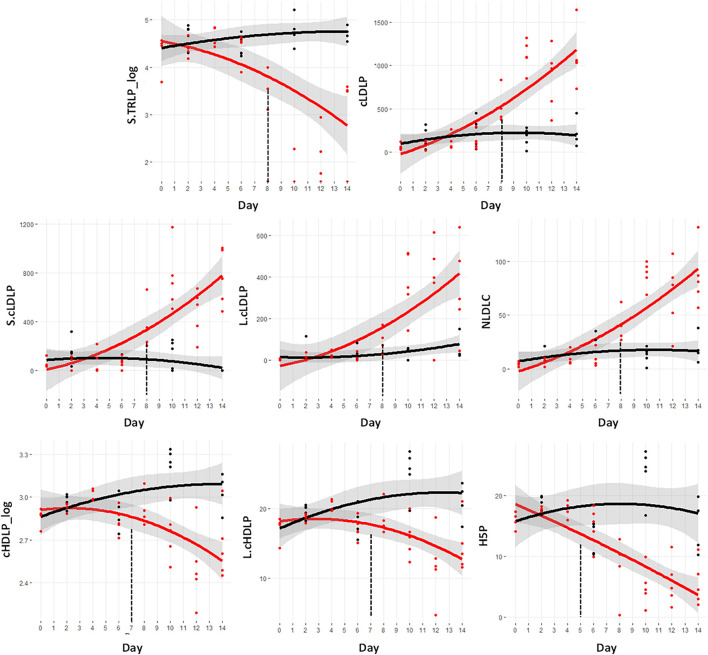
Trajectory of lipoprotein measurements that were statistically significantly different prior to the significant weight loss at day 9. Black indicates control mice, red indicates cachectic mice, and the vertical dashed lines indicate the day in which the data were first significantly different. S.TRLP, small triglyceride rich particles (i.e., very low density lipoproteins); cLDLP, calibrated low density lipoprotein particles (nmol/L); S.cLDLP, small calibrated low density lipoprotein particles (nmol/L); L.cLDLP, large calibrated low density lipoprotein particles (nmol/L); NLDLC, cholesterol fraction of LDL particles (mg/dL); cHDLP_log, log transformed values of the calibrated total high density lipoprotein particles (μmol/L); L.cHDLP, large calibrated high density lipoprotein particles (μmol/L); HP5, cHDLP subspecies with particle size = 10.3 nm (μmol/L).

Of the lipoprotein measurements, eight demonstrated significant changes prior to day 9. Figure 5 shows the metabolite trajectories of these lipoproteins. The VLDL particles in the assay are termed triglyceride rich lipoprotein particles (TRLP). The subfraction known as small TRLP (S.TRLP_log) was different at day 8. The total LDL particle numbers, designated cLDLP showed a significant increase at day 8, as did the small (S.cLDLP) and large (L.cLDLP) subfractions. The measurement of the cholesterol fraction of the LDL particles, termed NLDLC, was also significantly increased.

In contrast to the increases in LDL particles, there was a significant decrease in the HDL particles including the total particle number, cHDLP_log, and the large HDL particle subclass, L.cHDLP at day 7. The specific HDL subspecies known as H5P was the earliest lipoprotein marker of cachexia being different from controls at day 5 and dramatically declining through day 14.

#### Alterations in Phospholipids

The MS platform measured a total of 105 phospholipid species including 80 PCs, 15 lysophosphocholines (LPCs), and 10 SMs. This method cannot differentiate the individual fatty acids connected to the PCs or SM backbones. The nomenclature includes the total number of carbons in all of the fatty acid chains followed by the number of sites of unsaturation. For example, PC.aa.C42.3 indicates a total of 42 carbons in both fatty acid chains including a total of 3 sites of unsaturation. The “aa” indicates that both fatty acid chains are linked by ester bonds while “ae” indicates 1 ester and 1 ether linkage.

**FIGURE 6:**
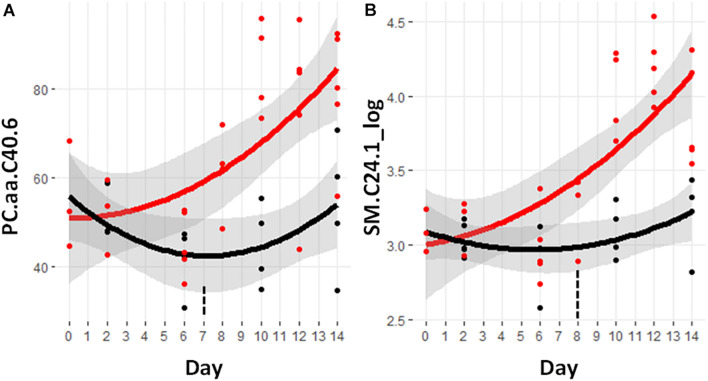
Trajectory of lipid measurements that were statistically significantly different prior to the significant weight loss at day 9. **(A)** PC.aa.C40.6, phosphocholine with a total of 40 carbons in the two fatty acid side chains attached by ester bonds and having six sites of unsaturation. **(B)** SM.C24.1, sphingomyelin containing a fatty acid containing 24 carbons and one site of unsaturation. Black indicates control mice, red indicates cachectic mice, and the vertical dashed lines indicate the day in which the data were first significantly different.

Of these lipids, a set of 57 metabolites demonstrated a significant difference between the control and cachectic groups in at least 1 day. Figure 3B shows the heatmap of *p*-values for these differences and only two of these are detectable before day 9. These are the glycerophosphocholine PC.aa.C40.6 and the sphingomyelin SM.C24.1_log; trajectories are shown in Figure 6. Most of the phosphocholines (including lysoPCs) are reduced with cachexia while the sphingomyelins are increased with cachexia. For the purpose of early detection, it appears that the changes in these types of lipids may not be useful.

#### Alterations in Markers of Oxidative Stress and Inflammation

**FIGURE 7:**
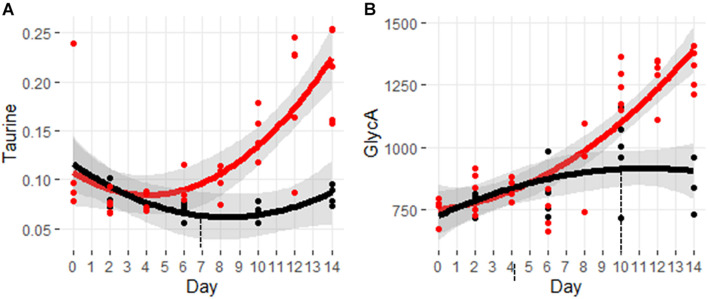
Trajectory of markers for oxidative stress and inflammation. **(A)** Taurine and **(B)** GlycA. Black indicates control mice, red indicates cachectic mice, and the vertical dashed lines indicate the day in which the data were first significantly different.

Taurine is a non-proteogenic sulfur amino acid that is abundant in the cells of many tissues. Taurine has been associated with a diverse set of cellular processes including cell signaling, membrane fluidity, and antioxidant defense from stress responses (Huxtable, 1992). Figure 7A shows the significant increase in taurine beginning at day 7.

Figure 7B shows the trajectory for the inflammatory marker, GlycA. This marker is composed of the aggregate signals of *N*-acetyl glycan groups attached primarily to acute phase proteins (Otvos et al., 2015). Increased levels of GlycA have been associated with various inflammatory conditions including arthritis, coronary heart disease, and colorectal cancer (Ormseth et al., 2015; Chandler et al., 2016; Connelly et al., 2017). Despite the dogma that cachexia involves an exuberant inflammatory response, it is interesting to note that levels of GlyA are not significantly elevated until day 10.

## Discussion

The goal of this study was not to search merely for new metabolic biomarkers for the detection of cachexia, but for biomarkers that reveal the presence of cachexia at the earliest possible point; ideally before the observation of weight loss. In our model, significant body weight reduction compared to controls was observed starting on day 9. Reduction in the tibialis muscle was observed at the same time, but reductions in the gastrocnemius and quadriceps were observed 2 days prior, on day 7. This reduction in skeletal muscle supports the idea that metabolic biomarkers of cachexia can be found ahead of observed weight loss.

A significant feature of this study was the more frequent sampling of mice across the time course of cachectic progression. In balancing the desire for high resolution sampling with the need to be appropriately parsimonious with the number of mice used, we sacrificed the control mice at four timepoints, starting at day 2 with sampling every 4th day until day 12. As the control mice displayed the anticipated progression in body and muscle weights associated with normal growth this reduced sampling was sufficient to model the trajectories. The sampling of the tumor-bearing mice was twice as frequent so that the trajectory analysis would be better able to detect changes in body composition and metabolites. The use of linear regression modeling of the data enabled a statistically rigorous evaluation across the entire trajectory.

Another option to follow the cachectic trajectory would be serial sampling from individual mice. The challenge with this design is that the amount of serum required for the multi-platform metabolomics analysis would be lethal to the mice. Additionally, other tissues were collected from this study including skeletal muscle and liver to be analyzed in the future.

A significant advantage of this study was the use of a very comprehensive and quantitative set of analytical platforms. The NMR platform is inherently quantitative and provides valuable information on some of the higher concentration, hydrophilic metabolites. The disadvantage of NMR is that it suffers from a relatively low detection sensitivity. The targeted MS platform has much greater sensitivity, detecting metabolites at much lower concentrations. The addition of calibration standards enabled quantitative analysis of a large panel of metabolites (Eibl et al., 2008). The NMR-based lipoprotein platform also provides quantitative measurements on a range of lipoprotein particle concentrations and sizes.

Some NMR-based metabolomics analyses of cachexia, such as reported by Yang et al. (2018), have sought an untargeted profiling of serum metabolites which includes signals for three of the main lipoprotein classes, VLDL, LDL, and HDL. This requires that the samples maintain the macromolecular components which yield broad signals that hinders the accurate quantification of many of the small molecule metabolites. In our study we filtered out the macromolecules to increase the spectral resolution for the analysis of small molecule metabolites and then used a separate sample for lipoprotein analysis.

An intriguing and unexpected observation in our study was the drop in the number of significantly different metabolites in the later days as shown in Figure 2. As noted earlier, several of the mice sacrificed at day 12 appeared to have more severe cachexia than those sacrificed at day 14, but the differences in body weight and muscle mass are not significant. The metabolite data shown in the *p*-value heatmaps in Figure 2 and in the boxplots in Figures 4–7 display examples of metabolites that continue to diverge through day 14 and those where statistically significant differences are lost in the final days. This observation may suggest that metabolic perturbations associated with the final moribund state of the animals may contribute to the metabolic profiles of the animals at day 14. Future studies with more animals will be required to better evaluate this possibility.

A reduction in circulating amino acids has been observed in a number of pre-clinical and clinical studies of cancer cachexia (Beck and Tisdale, 1989; O’Connell et al., 2008; Der-Torossian et al., 2013). This reduction, especially for the BCAAs, has been suggested to be due to increased proteolysis along with increased oxidation (Pin et al., 2019). In contrast, under starvation conditions, an elevation of circulating amino acids is observed (Pozefsky et al., 1976). Under these circumstances, muscle proteolysis releases amino acids into the circulation, which are then sent to the liver for gluconeogenesis. The BCAAs have been shown to increase in starvation along with a decrease in BCAA oxidation (Holecek et al., 2001). It is possible, that some degree of starvation may be playing a role in the recovery of the levels of some of the amino acids, in line with previous evidence in favor of reduced food intake in animal models for the study of cancer cachexia (Costelli et al., 2006; Murphy et al., 2012; Michaelis et al., 2019a,b). In this way the apparent recovery of amino acids levels in this hypercatabolic state may result from some fraction of those amino acids being diverted away from oxidative metabolism and being released into the circulation for gluconeogenesis.

In an earlier study from our laboratory we compared C26 tumor bearing mice with mice on a starvation-type diet with a 40% reduction in calorie intake (Der-Torossian et al., 2013). Not surprisingly, the calorie restricted mice showed a significant increase in the serum levels of the ketone body, β-hydroxybutyrate. The lack of any detectable increase in β-hydroxybutyrate in this study suggests that the mice do not develop a frank state of starvation. In this case, the mice may experience a less severe reduction in calorie intake that stimulates a higher demand for gluconeogenesis without the switch to the production of ketones.

Ideally, our study design would have included monitoring of food intake or pair feeding to fully evaluate the potential role of anorexia on the muscle wasting. Pair feeding with the C26 model of cachexia, was carried out in a study by Murphy et al. (2012). They found that control mice pair-fed with severely cachectic C26 tumor-bearing mice had no significant difference in muscle mass compared with *ad libitum* fed control mice, despite reduced food intake. This suggests that the loss of muscle mass in C26 bearing tumor mice is primarily linked to the tumor growth.

Among the set of 12 amino acids that are reduced, only five are considered fully essential amino acids (Met, Thr, Leu, Ile, and Val). The essential amino acids cannot be synthesized by mammalian cells and both normal and cancerous tissues depend on exogenous supply. The distinct decrease is not likely to come from a difference in dietary intake and thus, in this context, they are likely being used either for energy from cachectic hypermetabolism or biosynthetic processes driven by the tumor.

The earliest amino acid to diverge from control levels is methionine. Methionine functions as a methyl donor in the *S*-adenosy-L-methionine (SAM) cycle leading to the generation of polyamines and the synthesis of cysteine and glutathione. Normal cells can function in the absence of methionine, if supplied with homocysteine, but cancer cells cannot (Halpern et al., 1974; Cavuoto and Fenech, 2012). In fact, the potential of dietary restriction of methionine as a therapeutic strategy in cancer was raised more than 60 years ago (Sugimura et al., 1959). The observed early reduction in methionine may be driven by the proliferative demands of the tumor. Methionine is a precursor of cysteine which is needed for the synthesis of glutathione. The reduction in methionine may therefore be the result of increased demands for glutathione by the host to combat tumor generated reactive oxygen species (Bansal and Simon, 2018).

The early depletion of the non-proteogenic amino acid ornithine was curious as this cannot come from skeletal muscle catabolism. Ornithine is a key intermediate in the urea cycle which is involved in the conversion of toxic ammonia to urea. Under conditions of amino acid catabolism, ammonia is produced that eventually requires ornithine to enter into the urea cycle to generate non-toxic urea for excretion. Glutamate and aspartate also participate in the urea cycle and thus their observed reductions may also be related to the detoxification of ammonia generated from amino acid catabolism. It is also possible that the reduction in ornithine is due to metabolic effects of the tumor. Ornithine is a precursor for the synthesis of polyamines and several studies have shown that polyamine levels can be elevated in different tumor types (Kaczmarek et al., 1987; Thomas and Thomas, 2003).

Another non-proteogenic amino acid that appears as an early indicator of cachexia is taurine. This sulfur amino acid is thought to play a role in many cellular processes including cell development, cell signaling, membrane stability, and antioxidant defense from stress response (Huxtable, 1992). Several studies have shown that oxidative stress is associated with the development of cachexia (Barreiro et al., 2005; Laviano et al., 2007). In this case, the increase in taurine may be an endogenous response to this stress.

All three of the BCAAs were found to be decreased prior to the observation of weight loss. The BCAAs are primarily catabolized in skeletal muscle and increased rates of BCAA oxidation have been observed in a range of conditions associated with cachexia, including sepsis, trauma, and treatment with endotoxin or tumor necrosis factor (TNF) (Holecek, 1996; Holecek et al., 1997, 2001). This increased oxidation is consistent with the decreased serum levels. The muscle derived BCAA can also be used as a fuel for tumor energy metabolism (Ananieva and Wilkinson, 2018). Unfortunately, the use of a reduction in serum BCAAs as a component of an early cachexia diagnostic may be challenging due to other factors that affect their circulating levels. In particular, obesity and diabetes have been shown to lead to an increase in circulating BCAA levels (Newgard et al., 2009). As insulin resistance is a common comorbidity with cancer cachexia (Honors and Kinzig, 2012) BCAA levels may be influenced in different directions in cancer patients.

Reduced serum carnitine levels have been observed in several human clinical studies of cancer-induced cachexia (Vinci et al., 2005; Malaguarnera et al., 2006). In omnivores, approximately 75% of carnitine is obtained from the diet and 25% from endogenous synthesis (Rebouche, 1991). The endogenous synthesis of carnitine depends upon the availability of the amino acid precursors lysine and methionine. Although no differences were observed in the levels of lysine throughout the study, the clear and early reduction in methionine may contribute to the carnitine reductions. The role of carnitine is to transport fatty acids into the mitochondria for oxidation. This deficit could contribute to a reduction in the overall level of fatty acid oxidation. In our previous study, we also observed a significant reduction in serum free carnitine (Pin et al., 2019). In that study a significant reduction in muscle pyruvate dehydrogenase activity, a gatekeeping enzyme complex for glucose oxidation was observed indicating an overall shift to glycolytic metabolism in cachectic muscle. The early reduction of carnitine levels may be among the first metabolic switches that influence the switch in cachectic skeletal muscle from oxidative to glycolytic metabolism.

Decreases in two other acylcarnitines, the C3 and C5 species were also observed in cachexia. A unique feature of the C3 and C5 acylcarnitines is that they can come from either fatty acid or BCAA oxidation. The early reduction in acylcarnitines would be consistent with an increased oxidative flux of these amino acids. Although we suspect an overall reduction in fatty acid oxidation in cachexia, the reduced levels of circulating BCAAs along with reductions in these two acylcarnitine species would be consistent with an increase in BCAA oxidation in muscle and potentially the tumor.

The cachectic mice also displayed a number of early alterations in lipoprotein metabolism, but it should be noted that there are some important differences between lipoprotein metabolism in humans and mice. In particular, mice do not possess the cholesterol ester transport protein (CETP) which is a key protein involved in the transfer of cholesterol esters from HDL particles to LDL particles. This deficiency likely plays a role in the observation of very low levels of LDL particles in the control mice.

Several reports have indicated a prominent role for lipid metabolism and triglyceride lipases in the initiation and progression of cancer cachexia (Argiles et al., 2007; Chung et al., 2011; Das et al., 2011; Fearon, 2011). In particular, a reduction in the activity of LPL has been suggested to be a factor in cancer cachexia. White adipose tissue imports fatty acids by catabolizing circulating lipoproteins into fatty acids *via* membrane bound LPL. Decreased activity of LPL has been found during tumor growth in mice (Obeid and Emery, 1993) as well as in patients suffering from gastric and colorectal carcinoma (Nomura et al., 1997). Reduced LPL activity might suggest an overall increase circulating triglycerides as well as VLDL and LDP. Consistent with this, we see an increase several measurements of LDLs including total LDL particles (cLDLP), small LDL particles (S.cLDLP), and large LDL particles (L.cLDLP). The cholesterol concentration of the LDL particles is also significantly increased as measured by the NLDLC parameter.

In our previous study, we observed a similar increase in circulating LDL particles and then measured the LDL receptor levels in muscle and liver (Pin et al., 2019). An upregulation of LDL receptor in the muscle and no change in the liver was found in the cachectic mice. Apparently reduced clearance by the muscle and liver are not factors in the increased circulating LDL levels.

The earliest alterations in the lipoproteins was observed in the HDL particles with the total HDL particles (cHDLP_log) and large HDL particle concentration (L.cHDLP) reduced at day 7 and the level of the H5P subspecies being reduced at day 5. The nature of this reduction is not clear but may involve reduced HDL particle synthesis in the liver. In a recent study by Zwickl et al. (2020), the LDL and HDL cholesterol levels were measured in a cohort of 61 control patients, 22 patients defined as pre-cachectic, and 38 defined as cachectic. They found reductions in both LDL and HDL cholesterol with cachexia, but the lowering of LDL cholesterol was strongly impacted by the use of lipid lowering drugs in the patients. Given the complexities of lipoprotein metabolism and the limitations of mouse models, further studies in humans will have to be carried out to further evaluate the potential role of lipoprotein particles as early indicators of cachexia.

A large panel of lipid species including lysophospholipids, phospholipids, and sphingomyelins were measured by the targeted MS platform. Most of the significant alterations were observed after day 9 and involved reduced levels, although several of the sphingomyelins were increased. Several recent studies have also shown decreased levels of certain lysophospholipids with cancer cachexia (Cala et al., 2018; Miller et al., 2019; Morigny et al., 2020). In the paper by Morigny et al. (2020), a very comprehensive lipidomics analysis further found that high levels of sphingolipids, specifically ceramides and modified ceramides were associated with the progression of cachexia in several murine cachexia models and in human gastrointestinal cancer patients. We also found an increase in sphingolipids but with only one being elevated prior to observable weight loss. Despite the lack of early changes in sphingolipids in our specific model, the consistent observation of increased sphingolipids in other models suggest that these lipids warrant further study as diagnostic and potentially prognostic biomarkers for cachexia.

Inflammation is a hallmark of cachexia (De Matos-Neto et al., 2015; Seelaender et al., 2015). As part of the NMR-based lipoprotein analysis the inflammatory marker GlycA was measured. This marker has been associated with a number of inflammatory conditions including arthritis, coronary heart disease, and colorectal cancer (Ormseth et al., 2015; Chandler et al., 2016; Connelly et al., 2017). Tumor derived pro-inflammatory cytokines including TNF, interferon-γ (IFN-γ), and several interleukins (IL-6, IL-1β) have been associated with the development and progression of cachexia (Fearon et al., 2012). An increase in IL-6 is a well-established characteristic of the C26 model of cachexia (Bonetto et al., 2011, 2012; White et al., 2013). Given the role that inflammation plays in cachexia, it was somewhat surprising that GlycA was not elevated until day 10. The other conditions for which GlycA elevations are observed are more chronic conditions and therefore the late onset may simply be a function of the short timeframe of this model. In patients with a much longer time course, an inflammatory marker such as GlycA may be a useful component of a diagnostic panel.

## Conclusion

The results of this study demonstrate that metabolic biomarkers of cancer-induced cachexia can be detected several days in advance of the detection of weight loss. The decrease in amino acids levels was not an unprecedented observation, but the fact that a significant number of them were decreased 4–5 days prior to observable weight loss, at a point where the tumor would not even be palpable was indeed surprising. This study also suggests that some biomarkers of cachexia may be compromised at the later stages due to potentially competing mechanisms associated with the moribund status of the animals in the final days. The other metabolite species including acylcarnitines and lipoproteins also point to the early alterations in systemic metabolism during the initial establishment of the tumor.

Cachexia is indeed a systemic disorder, leading to dysregulations in a range of organ systems. Our previous study with the C26 tumor model found significant metabolic perturbations in muscle and liver in addition to the serum (Pin et al., 2019). Given the interplay between tumor derived factors and systemic dysregulation, it is challenging to identify metabolites that are specifically related to the muscle wasting and those that are driven by the demands of the tumor. It is likely that some metabolites are playing a role in both. As described earlier, the catabolism of BCAAs from the muscle may be oxidized in the cachectic muscle or may be shuttled to the tumor to support its energetic demands. In an earlier study from our laboratory, comparison of the metabolome of mice bearing the C26 tumor was compared to those bearing a non-cachexia inducing P388 lymphoma tumor. In that case the circulating metabolome of the P388 mice was much closer to the controls than the cachectic tumor bearing mice. Animals in these two groups had similar tumor burdens. The details and magnitude of muscle and tumor metabolic dysregulation are likely to vary for different tumor types. The application of metabolomics analyses to circulating biofluids will not have the resolution to deconvolve these different effects, but this approach holds significant promise for the early detection of metabolic dysregulation associated with cancer-induced cachexia. This early detection could help guide the development of new therapeutic interventions that will help maintain muscle mass, enabling continued treatment of the tumor and hopefully extending survival.

## Data Availability Statement

The original contributions presented in the study are included in the article/Supplementary Material, further inquiries can be directed to the corresponding author.

## Ethics Statement

All animal experiments were conducted with the approval of the Institutional Animal Care and Use Committee at Indiana University School of Medicine and were in compliance with the National Institutes of Health Guidelines for Use and Care of Laboratory Animals and with the ethical standards laid down in the 1964 Declaration of Helsinki and its later amendments.

## Author Contributions

TO’C and AB conceived and designed the experiments. RB, FP, and AB performed the *in vivo* experiments. TO’C analyzed the metabolomics data. LG-A, SD, and TO’C developed the statistical approaches and analyzed the time course data. TO’C, AB, and MC wrote and edited the manuscript. All authors contributed to the article and approved the submitted version.

## Conflict of Interest

The authors declare that the research was conducted in the absence of any commercial or financial relationships that could be construed as a potential conflict of interest.

## Publisher’s Note

All claims expressed in this article are solely those of the authors and do not necessarily represent those of their affiliated organizations, or those of the publisher, the editors and the reviewers. Any product that may be evaluated in this article, or claim that may be made by its manufacturer, is not guaranteed or endorsed by the publisher.

## Supplementary Material

Supplementary Figure 1 Boxplots for body weight, tumor volume, and muscle weights in the tumor bearing mice at days 10, 12, and 14. The body weight of the mice at day 12 are significantly lower than at day 10 but not statistically different from day 14. The tumor volumes as well as the muscle weights (gastrocnemius, tibialis, and quadriceps) are not significantly different in these last three timepoints suggesting a similar degree of cachexia.

Supplementary Table 1 *p*-Values for the differences between relative body weight and skeletal muscles in control versus cachectic mice. Red indicates a *p*-values < 0.05. No significant differences were observed prior to day 7.

Supplementary Table 2 Estimates and standard errors of the metabolites at each day from the quadratic model. Control_ and C26_Estimate, estimated means for the metabolite concentrations; Control_ and C26_StError, estimated standard errors for the metabolite concentrations; Diff_Estimate, difference of the estimated means between Control and C26 groups at each timepoint; Diff_StError, standard errors of the difference between the Control and C26 groups at each timepoint; *t*-stat, *T*-statistic from pairwise comparison of means of Control and C26 groups at each timepoint; *p*-values, *p*-values from pairwise comparison of means of Control and C26 groups at each timepoint.
