# Supplementary figures and images for: Metabolic Biomarkers for the Early Detection of Cancer Cachexia
Source: Front Cell Dev Biol. 2021 Sep 21;9:720096. doi: 10.3389/fcell.2021.720096 (PMC8490779; doi:10.3389/fcell.2021.720096)

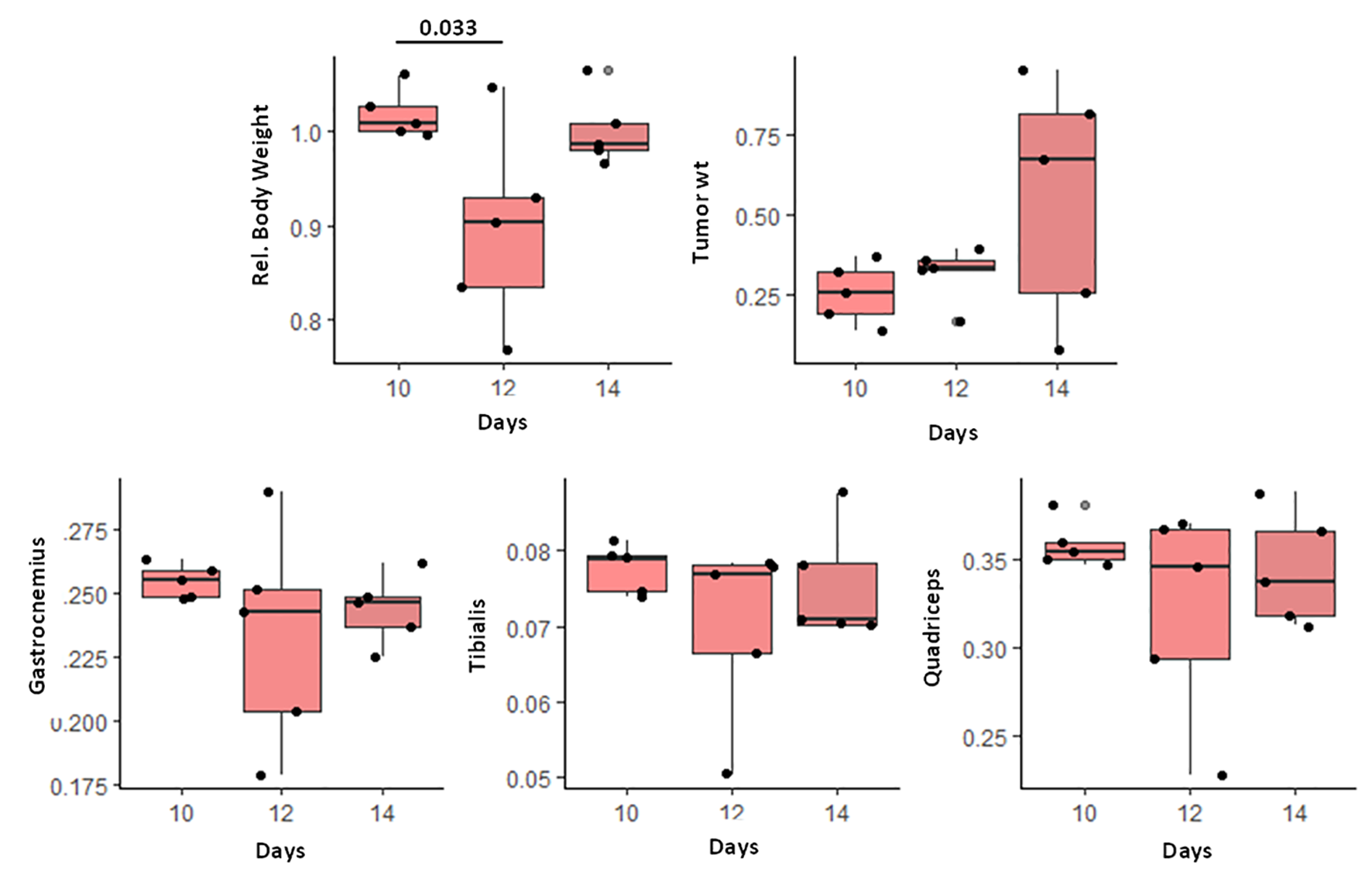

Supplement: Supplementary Figure 1 — Boxplots for body weight, tumor volume, and muscle weights in the tumor bearing mice at days 10, 12, and 14. The body weight of the mice at day 12 are significantly lower than at day 10 but not statistically different from day 14. The tumor volumes as well as the muscle weights (gastrocnemius, tibialis, and quadriceps) are not significantly different in these last three timepoints suggesting a similar degree of cachexia. [file Image_1.tif]
